# Valence and Core Photoelectron Spectra of Aqueous $I_{3}^{-}$ from Multi-Reference Quantum Chemistry

**DOI:** 10.3390/molecules28145319

**Published:** 2023-07-10

**Authors:** Vladislav Kochetov, Md Sabbir Ahsan, Dennis Hein, Iain Wilkinson, Sergey I. Bokarev

**Affiliations:** 1 Institut für Physik, Universität Rostock, Albert-Einstein-Str. 23-24, D-18059 Rostock, Germany; 2 Helmholtz-Zentrum Berlin für Materialien und Energie, Hahn-Meitner-Platz 1, D-14109 Berlin, Germany; 3 Department of Physics, Freie Universität Berlin, Arnimallee 14, D-14195 Berlin, Germany; 4 Operando Interfacial Photochemistry, Helmholtz-Zentrum Berlin für Materialien und Energie, Hahn-Meitner-Platz 1, D-14109 Berlin, Germany; 5 Department of Physics, Humboldt-Universität zu Berlin, Newtonstrasse 15, D-12489 Berlin, Germany; 6 Chemistry Department, School of Natural Sciences, Technische Universität München, Lichtenbergstr. 4, D-85748 Garching, Germany

**Keywords:** photoelectron spectra, shake-up bands, multi-reference methods, triiodide anion

## Abstract

The I${}_{3}^{-}$ molecule is known to undergo substantial structural reorganization upon solvation by a protic solvent, e.g., water. However, the details of this process are still controversially discussed in the literature. In the present study, we combined experimental and theoretical efforts to disentangle this controversy. The valence (5p), N${}_{4,5}$ (4d), and M${}_{4,5}$ (3d) edge photoelectron spectra were measured in an aqueous solution and computed using high-level multi-reference methods. Our previous publication mainly focused on obtaining reliable experimental evidence, whereas in the present article, we focused primarily on theoretical aspects. The complex electronic structure of I${}_{3}^{-}$ requires the inclusion of both static and dynamic correlation, e.g., via the multi-configurational perturbation theory treatment. However, the resulting photoelectron spectra appear to be very sensitive to problems with variational stability and intruder states. We attempted to obtain artifact-free spectra, allowing for a more reliable interpretation of experiments. Finally, we concluded that the 3d Photoelectron Spectrum (PES) is particularly informative, evidencing an almost linear structure with a smaller degree of bond asymmetry than previously reported.

## 1. Introduction

Photoelectron spectroscopy has proved itself to be a general and powerful tool to probe electronic structure in different states of aggregation [1]. For instance, in the liquid phase, the information obtained about energy levels on an absolute energy scale can be used to address solute–solvent interactions [2] thanks to the development of the liquid microjet technique [3,4]. Along with the main ionizing transitions, the correlation satellites, Shake-Ups (SUs), provide additional information on electron correlation in the studied system and on chemical bonding, since these transitions involve frontier orbitals. PES can be also used to indirectly infer the geometric structure of the studied species [5,6]. Particular success can be achieved by tandem experiments and theoretical calculations, with their synergy stemming from a mutual relationship between approaches. On the one hand, the theory provides invaluable guidance while interpreting experiments. The latter, in turn, are mandatory for benchmarking theoretical models and ensuring their accuracy.

In the present article, we employed such a tandem approach to attack the longstanding problem of the structure of the I${}_{3}^{-}$ molecule in solution. I${}_{3}^{-}$ is a remarkable molecule as it serves as a model system to study solvent-induced geometrical changes [7,8,9] and photo-fragmentation dynamics both in the gas and solution phases [10,11,12,13] and is used as an electrolyte in dye-sensitized solar cells [14,15,16]. The first of these qualities is the focus of the present investigation, where we attempted to provide more complete and reliable information about its fluxional structure in aqueous (and non-aqueous) solution [7,17,18], which still triggers debates in the literature and no consensus has yet been reached, see, e.g., Ref. [19] and references therein. Concisely, upon solvation, the linear centrosymmetric gas phase molecule [20] lowers its symmetry in protic solvents via elongating one of the I–I bonds and bending [8,9,17,18,21]. This symmetry breaking increases with the ascending proticity of the solvent, e.g., in the row ethanol-methanol-water. Although the principle mechanism is generally accepted for different solvents, the details provided by different investigations vary. For instance, hard X-ray scattering experiments that were performed following photoexcitation indirectly deliver the ground-state structure [18,22], leading to a conclusion that the molecule is notably bent (bond angle of 153°) with a large difference of the bond lengths, exceeding 0.4 Å. Previous theoretical simulations also support a broad distribution of molecular parameters but with a significantly smaller average angle of 170° [9,18]. Remarkably, the 4d PES was also applied to this system [8,23]; however, the signal-to-noise ratio or masking due to counterions and the broad, overlapping bands complicated the analysis for the aqueous solution sample. In the first part of our study [19], we presented the results of a joint experimental/theoretical investigation using X-ray liquid-jet PES in aqueous solution with a focus on the interpretation of the experiments. For instance, from the X-ray PES from different edges, we have concluded that the structure is closer to linear than it follows from the aforementioned X-ray scattering experiments [18,22]. In the present article, we put emphasis on the theoretical study, as a reliable description of this system is rather challenging and, in our opinion, deserves further, separate discussion.

In comparison to previous experimental and theoretical works, this study has two notable advantages: First of all, from the experimental viewpoint, a more careful subtraction of the background signal has been performed. This is crucial since the intensity ratios play an important role in the analysis of the PES results [8,19,21,23]. Moreover, here we focused not only on valence or 4d PES but also studied the 3d edge, which contains more information due to larger Spin–Orbit Coupling (SOC) splitting and non-overlapping bands. Second, from the computational viewpoint, static and dynamic electron correlation appear to be mandatory to study I${}_{3}^{-}$ [24]. Therefore, a proper choice of the active space [25] and correlation treatment, e.g., using multi-reference perturbation theory [26], is required. However, the application of multi-reference methods is prone to problems due to the variational stability of the active space and the sometimes poor convergence of the perturbation series, the so-called intruder states problem [27,28,29]. The thorough analysis reported here allowed us to obtain artifact-free theoretical spectra, having more predictive power for the interpretation of experiments.

Problems with computational methods prevented us from performing automated calculations for distributions extracted from molecular dynamics (MD) simulations containing many points, since the correctness of every calculation has to be thoroughly challenged by manual inspection. However, a library of structures from previous MD studies [8,23] has been used along with a systematic potential energy surface scan to aid in the sampling of the relevant geometric structural configuration space. Furthermore, to account for solvent effects, we employed the implicit solvation Polarized Continuum Model (PCM), accounting for the electronic response of the system to solvation. The hydrogen-bond network was accounted for by considering different geometric structures themselves and no explicit solvent molecules were considered. This approach, despite its seeming restrictiveness in terms of considered structures, has advantages over the protocols applied before as it is free from artifacts and provides a more reliable interpretation of experiments, see Section 3.4 below.

The article is organized as follows: first, in Section 2 we present the methodological aspects of the study. The molecular geometries entering our structural library as well as the systematic scan of the ground-state potential energy are described in Section 2.1. Details of the PES calculations and a brief description of experiments (for further information, see Ref. [19]) are given in Section 2.2 and Section 2.3, respectively. We start the discussion of the results in Section 3 with the general assignment of the valence, 4d, and 3d PES, Section 3.1, and then analyze the effect of geometric structure, Section 3.2, implicit solvation, Section 3.3, and nuances of electron correlation treatments, Section 3.4. The article ends with the discussion of the obtained results in view of the geometric structure of I${}_{3}^{-}$ in aqueous solution, Section 4, and conclusions, Section 5.

## 2. Methods

### 2.1. Considered Structures

Since the I${}_{3}^{-}$ ion is rather fluxional in solution, its structure is supposed to strongly depend on the local hydrogen-bond network in a protic polar solvent. Although studying the ground state solvation dynamics requires extensive sampling of structures using ab initio molecular dynamics—such as performed, e.g., in Refs. [8,9]—in this work, we conducted a series of single-point (static) calculations only. The reason for this choice is the high sensitivity of the results to computational details. For instance, the “fragile” composition of the orbital active space and numerous intruder state problems of the perturbational dynamic correlation treatment prevent us from relying on automated calculations along an MD trajectory. Even though the computations were based on a library of structures that lack generality, the human-controlled quality of the electronic structure calculations allowed us to avoid some artifacts, from which earlier works have suffered, as discussed in Section 3.4.

**Figure 1:**
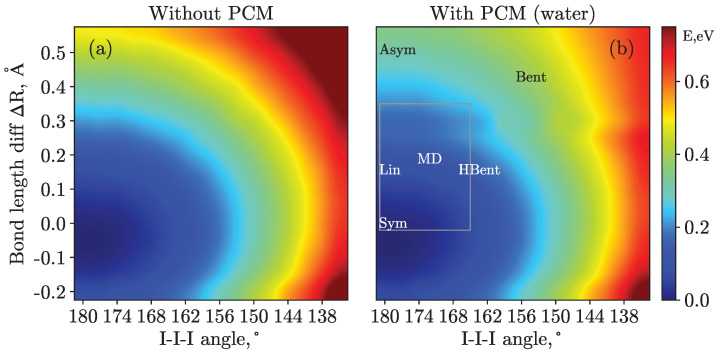
Potential energy surfaces calculated at the CASPT2 level of theory within the Val_Full active space without (panel (**a**)) and with (panel (**b**)) a PCM. Overall, 256 geometries were considered with 16 different I–I–I angles from 135° to 180° and 16 different I–I bond length differences, ΔR, varying from −0.15 Å to 0.6 Å. The colorbar values are given in eV relative to the potential energy minimum, which is linear symmetric in both cases. The six structures considered for the PES calculations are given in panel (**b**). Note that the fixed bond length differs from 2.93 Å in these cases. The gray rectangle displays the region of the systematic scan, see the main body of the text for futher details.

The six representative structures used in this work were chosen to represent different regions of the ground-state potential energy surface, see Figure 1b. The selection also accounted for the geometries often mentioned in the literature, either obtained experimentally or inferred from the extensive computational studies involving molecular dynamics simulations. These structures are summarized in Table 1. Essentially, they differ in the degree of asymmetry in I–I bond lengths and the bending angle, which is often considered to be the main parameter of symmetry breaking [30], being decisive for the system’s photophysics and photochemistry.

First of all, the fully symmetric structure ($D_{\infty h}$ point symmetry), labeled as Sym, was chosen. This structure was optimized in Ref. [23] with the Complete Active Space Second-Order Perturbation Theory (CASPT2) method without any solvent. Thus, it can be considered a theoretical equilibrium of the gas-phase structure of the ion.

The second structure, labeled Asym  represents the linear molecule with the $C_{\infty v}$ point group symmetry and the maximum difference in the I–I bond length, which has been extracted from the MD simulations [8] as that having the largest asymmetric stretch. Both the Sym and Asym structures have been used before to theoretically study the N${}_{4,5}$ PES of I in I${}_{3}^{-}$ [21], implying no symmetry restrictions.

Another geometry is denoted as MD, which does not exactly coincide but is very close to the averaged geometry, sampled by Jena et al. [9] from MD simulations, having an intermediate angle of 170°. It is also close to the geometry calculated using DFT with explicit inclusion of water molecules in the work of Kim et al. [18]

We also used two structures denoted as Lin and Bent as reference points for experimentally reported structures in methanol and water solvents [18], respectively. The first one is linear with $C_{\infty v}$ point symmetry and only a slight difference in the two bond lengths. The bond lengths given in Ref. [18] for methanol are 2.94 and 3.03 ± 0.04 Å; here, we have taken a slightly larger value of 3.09 Å to increase the difference from the Sym geometry. The second structure is bent ($C_{s}$ point symmetry) with a bond angle of 153°. The difference in the bond lengths is comparable to that of the Asym structure.

An intermediate bending angle was also included for a better sampling of the potential energy surface. The HBent structure has an intermediate angle of 165° but the bond lengths are very close to those of the Lin structure. As displayed in Figure 1b, the HBent position on the potential energy surface complements the triangular region of the considered geometries.

The ground-state potential energy surface was calculated over 256 geometries with 16 different I–I–I central angles from 135° to 180° and 16 different I–I bond lengths from 2.75 Å to 3.5 Å and is shown in Figure 1. The other I–I bond length was fixed at 2.93 Å, in accordance with previous reports [18]. The influence of the PCM solvent on the ground-state potential energy surface is discussed in Section 3.3.

Further, armed with the knowledge obtained from the structural library, we performed a systematic scan over the most important region of the potential energy surface. With this, we obtained a finer sampling of possible geometries that may underlay the 3d spectra, which are most sensitive to geometry variations. For the scan, the region corresponding to angles from 165° to 180° with a step of 1° (15 points) and bond length differences from 0.05 to 0.35 Å with 0.05 Å step (7 points) was selected. For instance, see the discussion of M${}_{4,5}$-edge PES in Section 3.2 and Figure 1b.

### 2.2. Multi-Reference Calculations

All the calculations were performed with the OpenMolcas [31]. The photoelectron spectra were estimated at the sudden approximation level as the squared norms of Dyson orbitals [32]. It is suitable for high photoelectron kinetic energies—such as those associated with the experimental photoelectron spectra considered here—and has been shown to be applicable for the study of I${}_{3}^{-}$ N${}_{4,5}$ PES spectra [21]. The ANO-RCC-TZP [33] basis set with the (22s19p13d5f3g)→[7s6p4d2f1g] contraction was chosen and scalar relativistic effects were accounted for via a Douglas–Kroll–Hess approach [34,35] within the second order perturbation theory framework. SOC is taken into account by the state interaction approach, making use of atomic mean-field integrals [36].

The previous study of the N${}_{4,5}$ PES of I${}_{3}^{-}$ [21] did not consider solvent effects. In the present study, the water environment was accounted for within a PCM treatment [37] for all considered spectra. The solute cavity was constructed by surrounding each atom with a sphere of radius 2.25 Å. See Figure S5 in the Supporting Information for an overview of the influence of the radii on the results. The slow component of the solvent response was calculated for the singlet initial state and was kept frozen for all final states of the ionized I${}_{3}^{•}$ molecule. For a more detailed discussion, see Section 3. The fast component of the response was specifically calculated for each state. It should be noted that the difference from conventional applications of PCM is that the excited state, in this case, is ionized, i.e., has a different charge than the initial state. However, implicit solvation does not account for the hydrogen bonds; the effect of the proticity of the solvent was taken into account solely by considering the different geometries of the molecule. It was shown that solvent-induced symmetry breaking of triiodide [17,38] is caused mainly by the redistribution of the negative charge and can be considered in terms of free energy surfaces, without explicit consideration of solvent–solute dynamics [39].

For each case, the Restricted Active Space Self–Consistent Field (RASSCF) calculation [40] was performed first. Several setups were used to calculate the valence, N${}_{4,5}$ and M${}_{4,5}$ core-excited state manifolds. The ground state wave function of I${}_{3}^{-}$ is single-configurational, with a main contribution of:

$$\ldots \;15\times {\left(3d\right)}^{2}\ldots \;15\times {\left(4d\right)}^{2}\ldots \;{\left(\sigma 5p_{z}\right)}^{2}{\left(\pi 5p_{x,y}\right)}^{4}{\left(n5p_{x,y}\right)}^{4}{\left(\pi^{*}5p_{x,y}\right)}^{4}{\left(n5p_{z}\right)}^{2}{\left(\sigma^{*}5p_{z}\right)}^{0}\;.$$

**Figure 2:**
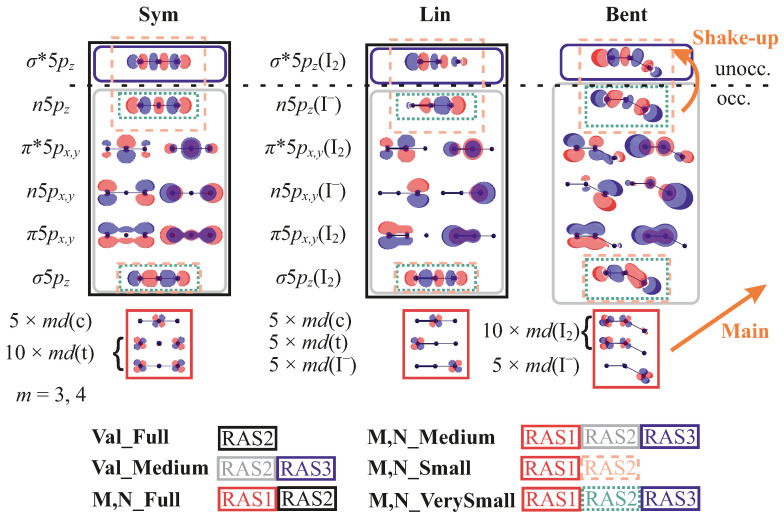
Active spaces used for the calculations of valence, M${}_{4,5}$, and N${}_{4,5}$-edge PES. The molecular orbitals (MOs) are given for three different geometries Sym, Lin, and Bent; for the other considered geometric structures, they are analogous to the closest case shown. Note that orbitals can be notably different for the molecule before and after ionization and for different spin multiplicities, nevertheless, they retain their qualitative character. Only three representative md orbitals (m=3,4) out of 15 are shown. They are classified as central (c) and terminal (t) atomic orbitals where possible. The other orbitals are provisionally classified as σ and π bonding and antibonding, as well as non-bonding (*n*). For the geometries where the molecule represents the I_2_—I^−^ case, they are alternatively subdivided on the I_2_ and I^−^ orbitals. The z-axis coincides with the symmetry axis of the molecule or of the I_2_ fragment. The main and SU transitions are also shown.

For the notation of the orbitals, see Figure 2. In the case of the Sym configuration of the molecule, one can additionally subdivide the 3d and 4d orbitals in the group of five belonging to the central I atom and in the group of ten representing combinations of orbitals residing on terminal atoms. The other orbitals are provisionally classified as σ and π bonding and antibonding as well as non-bonding (*n*). In the case of asymmetric structures, such as the Bent structure, the orbitals can be better described as those of the I${}_{2}$ molecule and I${}^{-}$ ion, forming an adduct, I${}_{2}$—I${}^{-}$. Note that orbitals can be notably different for the molecule before and after ionization and for different spin multiplicities, nevertheless, they retain their qualitative character, which we will refer to in the following.

In the valence PES, the main (1h) ionization transitions occur from the occupied 5p orbitals, and in M${}_{4,5}$ and N${}_{4,5}$ spectra, from the 3d and 4d orbitals, respectively. The correlation satellites, 2h1p SUs, are due to additional excitation from the occupied 5p-like orbitals to the unoccupied $\sigma^{*}5p_{z}$. The higher-order 3h2p SUs might also be possible. Having this in mind, the following active spaces were considered:

- Val_Full: All nine valence 5p orbitals, forming σ and π bonding and anti-bonding as well as non-bonding MOs, are included in the RAS2 subspace. Full CI (FCI) is performed within this subspace, see Figure 2. This active space allows the 3h2p configurations to be included in addition to the 1h and 2h1p ones, which are of main interest here. This active space can be also denoted as (16/15 e${}^{-}$; RAS1: 0, RAS2: 9, RAS3:0; 0h, FCI, 0p), meaning that the ionized states have 15 electrons instead of 16.
- Val_Medium: It is essentially the same as Val_Full but the $\sigma^{*}5p_{z}$ orbital was put to the RAS3 space, allowing for only one electron there, i.e., (16/15 e${}^{-}$; RAS1: 0, RAS2: 8, RAS3:1; 0h, FCI, 1p). Effectively, since only doubly-occupied orbitals enter RAS2, it only includes 1h and 2h1p configurations. This active space allows the reproduction of the key features of the valence spectrum but with lower effort.
- N_Full: To access the N${}_{4,5}$-edges, the fifteen 4d orbitals were put into the RAS1 space allowing for a single 4d hole. The rest of the active space is the same as in Val_Full, thus making it a (46/45 e${}^{-}$; RAS1: 15, RAS2: 9, RAS3:0; 1h, FCI, 0p) space. The results for a similar active space, M_Full, were obtained only for the 3d spectrum of the Sym structure with $D_{2h}$ symmetry without PCM. A large number of states and unstable Restricted Active Space Second-Order Perturbation Theory (RASPT2) calculations, that should be performed and verified for each irreducible representation, make the results extremely computationally demanding and unreliable. Thus, the associated results are not suitable for systematic comparison but are briefly discussed here for consistency.
- N_Medium: The reduction of the active space is the same as between Val_Full and Val_Medium resulting in a (46/45 e${}^{-}$; RAS1: 15, RAS2: 8, RAS3:1; 1h, FCI, 1p) space.
- N_Small: In this case, only three combinations $5p_{z}$ orbitals, $\sigma 5p_{z}$, $n5p_{z}$, and $\sigma^{*}5p_{z})$, were included into RAS2, constituting a (34/33 e${}^{-}$; RAS1: 15, RAS2: 3, RAS3:0; 1h, FCI, 0p) active space. This active space has been used before in Ref. [21] and includes only those orbitals for which the SU excitation has a “dipole-allowed” nature. Although this seems restrictive, it reproduces the features of N${}_{4,5}$-edge PES [21].
- N_VerySmall: This is a particularly small AS, where two occupied $p_{z}$ orbitals are put in RAS2 and the unoccupied $\sigma^{*}5p_{z}$ is placed in RAS3 with one electron allowed.
- M_Full:, M_Medium, M_Small The active spaces for the M${}_{4,5}$-edge are full analogues of those for the N${}_{4,5}$-edge, with the difference that instead of the 4d orbitals, they include the 3d ones.

For a pictorial representation of all active spaces, see Figure 2. The number of states calculated for each active space is given in Table 2.

To account for the major part of the dynamic correlation, the RASPT2 [41] calculations were performed on top of the RASSCF results. All orbitals from the K, L, M, and N shells were kept frozen apart from the 5s and 5p and active MOs. In some cases, the intruder state problem has manifested itself severely. The imaginary level shift [42] of 0.1 Hartree has been applied to valence calculations and 0.2 to core—spectra calculations, in addition to a standard IPEA shift of 0.25 Hartree. Loosened thresholds for the removal of zero-norm components and for the removal of near linear dependencies in the first-order perturbed wave function (THRESH keyword was set to $10^{-8}$ and $10^{-6}$, respectively) were set for core calculations to reach convergence. X Multi- State RASPT2 (XMS-RASPT2) [43], ordinary Multi-State RASPT2 (MS-RASPT2) [44], and Single-State RASPT2 (SS-RASPT2) calculations were performed.

For each spin manifold, the State-Averaged RASSCF calculations included all the states of interest. In all cases, only one singlet state was considered as a neutral initial state. The inclusion of low-lying triplet excited states to enable SOC did not appreciably affected the results and is not discussed further. The number of considered SOC-coupled core-excited doublet and quartet states of the ionized molecule, I${}_{3}^{•}$, for different active spaces are given in Table 2. For core spectra, the quartets were shown [21] to be of little importance, which has also been proven by our calculations. For the doublets, a few highest excited states were excluded that helped to solve intruder state problem in RASPT2. These states are mostly ascribed to double SU features, which never appear in experiments and often create problems for convergence of the calculations.

### 2.3. Experiments

The high-energy-resolution experiments were performed at the P04 undulator beamline at PETRA III [45] using the EASI instrument [46]. For more details, see Ref. [19]. For comparison, only the bulk-sensitive high-energy-resolution experiments on valence, I 4d, and I 3d edges were chosen from the variety of spectra presented in our previous work [19], measured with photon energies of 600 eV, 600 eV, and 1175 eV, respectively. Experimental spectra were obtained from samples with Na${}^{+}$ counter ions by mixing NaI and I${}_{2}$ solutions in water that allowed isolation and interpretation of the full 4d spectrum. Triiodide contributions to the spectra were isolated by Binding Energy (BE)-shifting [2,47,48], intensity-scaling, and subtracting sequentially-collected I${}_{3}^{-}$ background solution spectra from the mixed I${}_{3}^{-}$—I${}_{3}^{-}$ sample solution spectra. The associated iodide spectra scaling factors were determined via a series of UV-Vis absorption spectra analysis. Hence, no assumptions were made about the relative valence, I 4d, and I 3d ionization cross-sections.

## 3. Results

### 3.1. General PES Assignment

**Figure 3:**
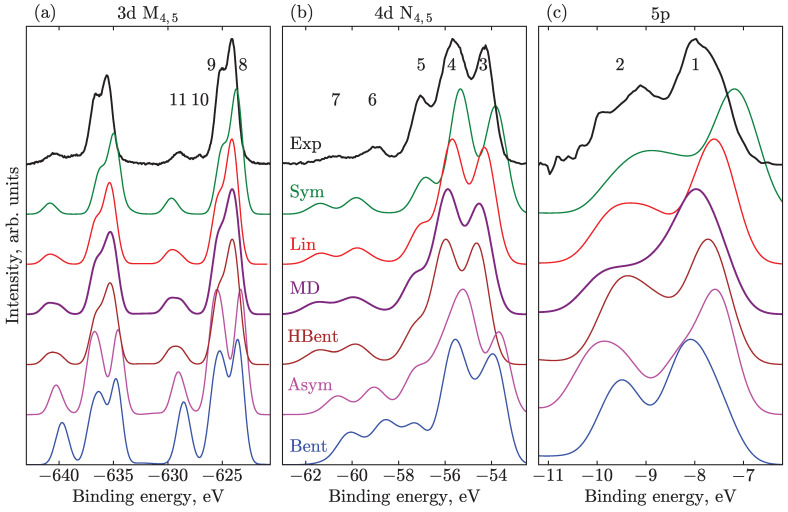
PES computed at the SS-RASPT2 level of theory for different geometries: (**a**) 3d with the M_Medium active space; overall shift of 6.5 eV is applied to all spectra, as well as Gaussian broadening of 1.25 eV FWHM. (**b**) 4d with the N_Medium space and no global shift; Gaussian broadening is 1.05 eV. (**c**) Valence 5p spectra with Val_Medium space, no shift, Gaussian broadening 0.85 eV. The curves are vertically shifted for visual clarity. The numbered bands are assigned in the main body of the text.

To facilitate the following discussion of the specific spectral features, we first introduce the characteristic PES bands, which are present in the experiment, and give an overall assignment. As can be seen from Figure 2, the important frontier MOs comprise different bonding, anti-bonding, and non-bonding combinations of the iodine 5p atomic orbitals. Depending on the symmetry of the molecule, they can be classified as delocalized over all three atoms or belonging to the I${}_{2}$ and I${}^{-}$ moieties. Additionally, for the symmetric case, one distinguishes orbitals residing predominantly on terminal (t) or central (i) iodine atoms. The ionization of an electron from the outer levels (eight highest occupied 5p-like orbitals) constitutes the main contribution to the valence PES (peaks 1 and 2 in Figure 3c).

The core 3d and 4d orbitals are notably more localized and can be, in most cases, ascribed to the central (c), terminal (t), or ionic (i) iodine atoms. The SU features, which serve as a quite sensitive probe of the geometric structure for this system, [19,21] originate mainly from the $n5p_{z}\to \sigma^{*}5p_{z}$ excitations accompanying the ionization. For lower symmetries, other occupied valence MOs containing $5p_{x,y}$ orbitals can also take part in the SU excitations to the $\sigma^{*}5p_{z}$ unoccupied valence orbitals. The main features, as well as the correlation satellites, are additionally split into $md_{5/2}$, lower BE, and $md_{3/2}$ (m=3,4), higher BE, components due to SOC. In the case of N${}_{4,5}$-edges, the experimental splitting is 1.5 eV. For the M${}_{4,5}$-edges, the splitting is 11.4 eV.

In the experimental N${}_{4,5}$-edge PES, Figure 3, the three bands that occur at about 54.2 eV, 55.6 eV, and 57.1 eV (peaks 3, 4, 5, respectively) can be attributed to the 4d${}_{5/2}$ ionization of the I${}^{-}$-like (i) atom, the overlapping terminal 4d${}_{5/2}$(t), central 4d${}_{5/2}$(c), and ionic 4d${}_{3/2}$(i), and overlapping 4d${}_{3/2}$(t) and 4d${}_{3/2}$(c) ionization channels, respectively. The remaining peaks at 59.0 eV and 60.8 eV (peaks 6 and 7) are primarily assigned to the SU ionization of mixed character; however, the main contribution can be identified as $n5p_{z}\to \sigma^{*}5p_{z}$. The splitting between these two features is about 1.7 eV mainly due to SOC splitting of the ionized 4d${}_{5/2}$ and 4d${}_{3/2}$ atomic energy levels.

The previous theoretical study [21] reported a couple of extra charge-transfer features in N${}_{4,5}$-edge spectrum in an I${}_{3}^{-}$ asymmetric molecule (Asym). These features could be notable fingerprints of the presumably more asymmetric structure of aqueous triiodide. However, upon detailed analysis, we have found neither experimental nor theoretical evidence for additional core-level ionization-induced charge-transfer processes in the vicinity of 52 eV BE, as discussed in Reference [21]. Similarly, we do not observe any signatures of SU processes involving excitation from higher BE valence orbitals, specifically in the BE region spanning 63–70 eV. For more discussion of this issue in view of the intruder states problem, see Section 3.4.

The experimental 3d spectrum shows the same pattern as the 4d. However, the features are more well separated due to stronger SOC. The features with BEs of 624.12 eV (peaks 8) and 625.22 (peak 9) correspond to 3d${}_{5/2}$(i/t) and 3d${}_{5/2}$(c) states, respectively, followed by a pair of SUs (peaks 10 and 11), which were tentatively ascribed in Ref. [19] to (c) and (i/t) $n5p_{z}\to \sigma^{*}5p_{z}$ SU process, leaving, however, the possibility for an alternative origin of these peaks. Peak 10 is of great interest because it has not appeared in the experiment for triiodide in ethanol solution. As the aqueous solution is supposed to make the molecule more asymmetric and thus allow for additional transitions, which are forbidden for more symmetric geometries, this feature is logically associated with an earlier unobserved central (c) SU. It was shown that peak 10 definitely points to the lower symmetry of aqueous triiodide compared to ethanol but there was no understanding of the associated extent. We will discuss the nature of these features in detail in Section 3.2 and Section 4 in connection to the geometrical structure of the ion.

As described elsewhere [19], the applied methodology reproduces the experimental PES quite well and allows for a straightforward interpretation of those results. As the details of the corresponding joint experimental and theoretical analysis are presented in the other article [19], here we focus primarily on the essential computational aspects.

### 3.2. Effect of Geometry

The geometric structure of the I${}_{3}^{-}$ molecule notably influences its valence, M${}_{4,5}$, N${}_{4,5}$, and valence PES, as illustrated in Figure 3 at the SS-RASPT2 level of theory. The valence and N${}_{4,5}$ spectra have not been shifted, while the M${}_{4,5}$ spectra are all globally shifted by the same value of 6.5 eV. This latter shift is caused by factors such as the lower quality of the basis set for deeper-lying core orbitals and the neglect of valence–core correlation. A uniform phenomenological Gaussian broadening of 0.85 eV, 1.05 eV, and 1.25 eV is applied to valence, N${}_{4,5}$, and M${}_{4,5}$ transitions, respectively, for convenience of comparison to the experiment.

Previously, the variations in the positions and heights of the PES peaks in different solvents have been connected to the lowering of the I${}_{3}^{-}$ solute symmetry [8,9,21]. We note that the symmetry of the molecule is sufficiently lowered from $D_{\infty h}$ for the Sym structure to $C_{\infty v}$ for the Lin and Asym geometries, and to $C_{s}$ for the Bent structures. One can approximately arrange the configurations in a row according to the decreasing symmetry: Sym, Lin, MD, HBent, Asym, and Bent. A natural effect of such lowering is degeneracy lifting, leading to broader peaks. Moreover, some symmetry selection rules get lifted for the SU transitions, that is, more of them become allowed. For instance, the ionization from the 3d(c) and 4d(c) orbitals residing on the central I atom (see Figure 2) and simultaneous excitation from the $n5p_{z}$ to the $\sigma^{*}5p_{z}$ orbitals is forbidden for the Sym geometry [49], but becomes allowed for the other structures. In general, one can expect an overall increase in the intensity of the SU features with decreasing symmetry of the structure, as discussed below. (However, note that strictly speaking, photoelectron transitions do not obey any symmetry selection rules as in the continuum one can always find a free photoelectron wavefunction of suitable symmetry. Therefore, the statement about lifting selection rules and SUs gaining allowed nature only holds within the sudden approximation, without considering conjugate Dyson orbitals).

Concerning the finer structure of the spectra, such as positions and intensities of prominent bands, the PES computed for different geometrical structures are substantially different. The least sensitive in this respect is the valence spectrum, where only slight variations are observed, see Figure 3c. The intensity distribution of the valence spectra in the shown BE range is not very informative about the geometry of I${}_{3}^{-}$ ions in aqueous solution. The less symmetric species lead to a slight overall blue shift in the BEs by about 1 eV. The best agreement in terms of peak intensities and positions is reached for the Lin, MD, and HBent geometries. The experiment does not cover the −15–−10 eV BE region due to the overlap with the water solvent spectrum, which dominates the total spectrum in this spectral region. However, in the computations, we have identified a number of peaks, which are more sensitive to speciation. These peaks represent multi-electron excitations, which presumably can be seen in experiments in other solvents or if improved water-solvent-signal background subtraction methodologies can be developed.

In the case of the N${}_{4,5}$ spectra in Figure 3b, one can also deduce a systematic blueshift. This observation is also supported by experiments when comparing ethanol and aqueous 4d triiodide spectra, with a relative solvent-induced shift of 0.5 eV. One observes slight shifts of the major three bands in the 53–58 eV range of BEs (peaks 3, 4, 5). The symmetric geometries—namely the Sym, Lin, and HBent—fit better to the intensity distribution of the first two main lines (at about −54 and −56 eV); but the position of SUs is in better agreement for species with larger bond differences—Asym and Bent geometries. The position of these bands almost coincides with experiments for the Bent geometry, whereas the others occur at smaller BEs. However, one should note that the position of SUs in the calculations is very sensitive to the dynamic correlation, with a tendency for the SU BEs to be underestimated, see Section 3.4. The SUs tend to shift to higher energies upon symmetry lowering. As can be expected, the intensity of the SUs in the less symmetrical Bent geometry is higher than in the Sym geometry since more transitions are allowed. As discussed in Ref. [19] in detail, the joint analysis of experimental and theoretical spectra evidences a predominantly linear geometry with a possible small fraction of bent structure in the ground state distribution. A broad bimodal nuclear distribution in the aqueous solution seems to be rather improbable; however, one can suppose that the valence, N${}_{4,5}$, and M${}_{4,5}$ PESs are explained best as a superposition of the Lin and Bent-like structures.

In Figure 3a the M${}_{4,5}$-edge spectra of the linear and near-linear structures—Sym, Lin, MD, and HBent—have very similar spectral shapes and the splitting due to ionization from the central and terminal and ionic 3d orbitals almost reproduces that of the experiment: cf. features at about 626 eV (peaks 8, 9) and 637 eV (not labeled). Besides this, the overall systematic shift of the main features in these structures is observed. The Sym structure is red shifted compared to the Lin structure by 0.5 eV; likewise, the experimental ethanol spectrum is red shifted with respect to aqueous one by 0.85 eV (i.e., the difference of solvent-induced BE shifts). In the Asym and Bent case, individual transitions are distributed such that they appear as separated bands, with the splitting between peaks 8 and 9 being much larger than the experimental splitting of 1.1 eV. The position of the central peak (peak 9) is rather insensitive to geometrical variations; the same can be seen for peak 5 in the N${}_{4,5}$ spectrum. The energetic positions of the core orbitals at the central iodine atom are less sensitive to geometrical and adjacent charge rearrangements than those on the side atoms (t and i). Upon bond elongation, the charge density is transferred from the (t)-side to (i)-side, while the charge on the central iodine atom stays essentially the same, as confirmed by studies on the N${}_{4,5}$-edge photoelectron signals [9]. The main 3d peak, 8, which includes the 3d(i) contribution, systematically shifts to larger BEs if the symmetry is lowered, since more electron density at the (i)-side makes the 3d(i)-electrons less strongly bound. Pulling the ionic iodine away from the other two, the splitting between peaks 8 and 9 starts to increase due to increasing separation between the (i) and (t) ionization energies. The same holds true for the N${}_{4,5}$ peaks, 3 and 4, which are also mostly due to ionization from the ionic and terminal atoms. In this case, the change is less pronounced because of the overlap between the SOC-split bands. It should be noted that the intensity ratio of peaks 8 and 9 is best reproduced by the MD geometry. Peak 9 is less intense in the Sym and Lin geometries with respect to the experiment, and the spectra calculated at these geometries have better agreement with the experiments in an ethanol solvent.

In the case of correlation satellites, one observes the same general trend as for the N${}_{4,5}$-edge PES: with lowering symmetry, the intensity of the SUs is increased, and the separation of the SUs and main features becomes smaller. However, due to the larger SOC splitting for the M${}_{4,5}$-edge, we can observe more details, i.e., splitting of the SU features. For the Sym geometry, there is a single SU band approximately at the position of peak 11. With symmetry lowering already at the Lin geometry, this feature becomes asymmetric. In a more detailed analysis of the stick spectra, one can clearly see that the satellites associated with ionization from the central atom (which are forbidden for Sym geometry) grow in intensity and shift to lower BEs. For the MD geometry, the splitting between the two groups of transitions results in an almost-two-headed SU, with a splitting of more than 1 eV, which is less, however, than the experimental splitting between peaks 10 and 11 of 1.7 eV.

**Figure 4:**
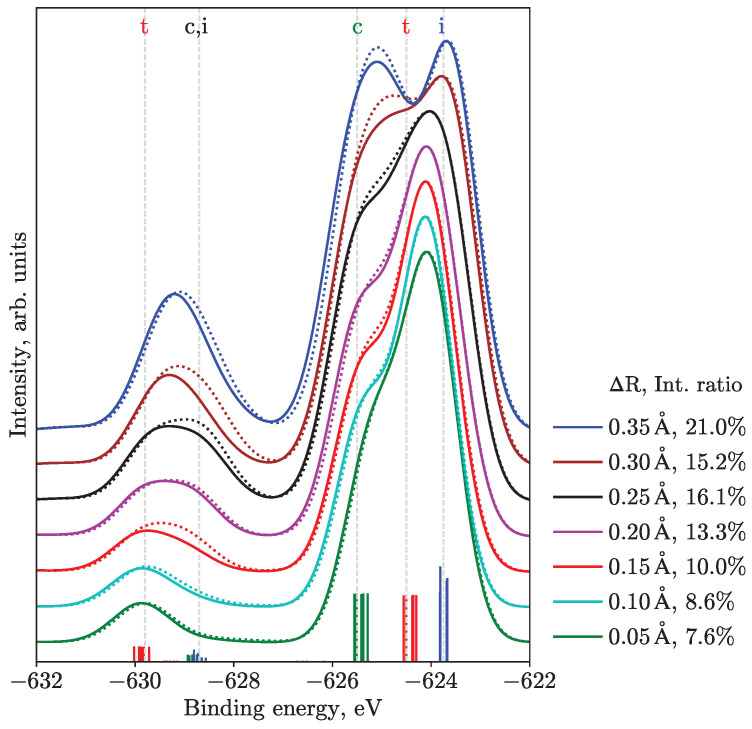
M${}_{5}$ PES calculated for linear (I–I–I angle of 180°, solid lines) and bent (165°, dotted lines) geometries and varied bond length difference ΔR. The legend shows the difference of bond lengths and SU-to-main intensity ratio. The experimental value for this ratio in aqueous solution is 12.2%. The stick spectra, as well as the eye-guiding gray lines, demonstrate groups of transitions for the MD structure, corresponding to ΔR = 0.20 Å. The color of the stick spectra relates to the central (c, green), terminal (t, red), and ionic (i, blue) atoms.

An additional quantity that discriminates between different geometries is the intensity ratio of the SU ionization features to the main transitions, building on the considerations of the lifting of degeneracy and symmetry selection rules. In particular, we performed calculations with varied bond length differences for the M${}_{5}$ edge, see Figure 4, where the intensity ratio is also given. During this scan, the shorter bond length was fixed at 2.93 Å. Increasing the asymmetry, i.e., increasing one of the bond lengths while keeping the other fixed, leads to higher integral intensities of SU ionization peaks. Remarkably, for a bond-length difference of 0.15–0.2 Å, the SU-to-main-peak intensity ratio (10–13.3%) is in agreement with the experiment (12.2%). One should notice that ΔR equal to 0.15 Å corresponds to the Lin geometry and one equal to 0.20 Å relates to the MD geometry. For further discussion on this observation, see Section 4, where we consider some peculiarities of the computational method.

### 3.3. Effect of PCM

The PCM accounts for the polarizability of the solvent and its induced charge rearrangement, which acts on the solute molecule and can lead, e.g., to a shift of its energy levels and changes in other properties. The strength of the solvent effects is supposed to grow in the series: ethanol (ε = 24.3), methanol (ε = 32.7), to water (ε = 78.4); here the values of the dielectric constant are given for 298 K. As discussed above, the main influence of the solvent is via stabilizing different geometric structures. While the Sym geometry has no permanent dipole moment, the asymmetric and especially bent structures are increasingly dipolar. From these simple considerations [50], an increasingly polar solvent should favor asymmetric and bent structures. However, the purely electronic response, i.e., due to solvent polarization at fixed geometry, is also notable.

The selection of the atomic radii that form the solute cavity may be crucial for the PCM treatment. However, we observed a rather stable spectral behavior, when varying the radii from 1.98 to 2.25 Å, see Figure S5 in the Supporting Information, representing the range between the van der Waals radius of the I atoms in different environments and its ionic radius [51]. Only at substantially larger radii, e.g., 2.5 Å, do the results notably differ from those with the more reasonable values in the aforementioned range. In the main text, we accordingly only consider results corresponding to 2.25 Å radii.

We start with a discussion of the PCM influence on the ground-state potential energy surface computed with the CASPT2 method and shown in Figure 1 for cases with and without solvent effects. Without PCM, i.e., in the gas phase, the potential has a single distinct minimum at a configuration with equal bond lengths and a bond angle of 180° (similar to the Sym structure). Including the solvent, the main minimum retains its position, and a clear flattening of potential energy surface is seen in the ΔR direction when applying a solvent (water) reaction field, as clearly seen in panel (b). Furthermore, a very shallow minimum can be identified at a ΔR of 0.3 Å. However, no clear plateau is seen in the direction of the bending angle, suggesting less sensitivity of the total energy to angle variations. Note also the stabilization of the minimum configuration by 1.8 eV by the PCM solvent relative to the gas phase. Although the present calculation results do not support a bimodal geometric structure distribution, i.e., two minima on the potential surface with an energy difference of the order of kT, they evidence a notable stabilization of linear asymmetric structures. In turn, they disfavor bent structures, a conclusion that is in line with the interpretation of the PES observables presented in Ref. [19] and in the present article. Nevertheless, a more realistic solvent treatment, e.g., explicitly including the first solvation shell, at the same level of theory (CASPT2) or higher might change the structure of the ground-state potential surface.

When considering the PES, the most persistent effect in all of the calculations, including PCM, is an overall shift to higher (more negative) BEs by about 3 eV due to the stabilization of the ground state of the negatively charged I${}_{3}^{-}$, while the final state I${}_{3}^{•}$ is uncharged and, thus, less affected. For most of the geometries, the PCM spectra agree with the experiment better and require less (or even no) global shift, except for the 3d spectra, where energies are generally obtained with larger error.

**Figure 5:**
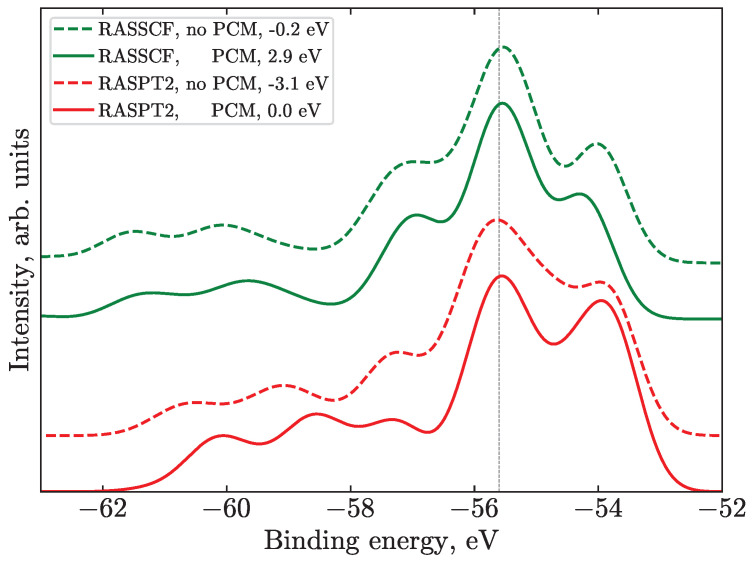
Illustration of the effect of the PCM on the N${}_{4,5}$ spectra for the Bent geometry at different levels of theory within the N_Medium active space. This geometry is chosen for comparison as it is the most asymmetric and thus most sensitive to PCM effects, since it possesses the largest dipole moment. The experimental spectrum is not shown, though all of the spectra in the figure are aligned at the position of the experimental peak 4 (vertical line). The related values of the simulated spectral shifts are given in the legend. The spectra are shifted vertically for clarity.

Upon inclusion of solvent effects, one can also observe a more intricate redistribution of intensities, which in some cases is caused by changing transition energies. This especially holds true for the SU features because they reflect the energies of the outer valence orbitals, which are substantially more sensitive to the environment than the core orbitals. In particular, the form and the main–SU energy splitting changes for the core spectra. This is illustrated for the N${}_{4,5}$ edge spectra shown in Figure 5.

While considering the influence of the PCM solvent, it is essential to analyze the dipole moment of the involved structure [50]. Therefore, from this viewpoint, the Bent geometry is especially interesting as it possesses the largest dipole moment. In this case, the PCM leads to a larger redistribution of intensity and even changes in the shape of main peaks, see Figure 5. Note that this is purely a solvent-response effect, as the geometry stays fixed. Other geometries and other edges also follow the trends observed for the Bent structure. The splitting between the (t), (i) ionization features on the one side and the (c) features on the other becomes smaller at the RASSCF level (green lines), which leads to a less prominent side peak/shoulder. At the RASPT2 level (red lines), the PCM also mainly influences the band at about 54 eV, changing its intensity. This can be explained by the charge stabilization at the ionic iodine; thus the solvent-induced BE shift of (i) is different from the (t) and (c) atom signals. Spectra calculated with the PCM (solid lines in Figure 5) are shifted by 3.1 eV to lower BEs in both cases, and the SUs are closer to the main lines by about 0.5 eV, which can be attributed to the LUMO $\sigma^{*}$ energy change.

The influence of the PCM on different types of spectra as well as different active spaces for linear and near-linear geometries is less pronounced because of the more symmetric charge distribution and lower value of the dipole moment. Although we do not present the results for all edges and geometries in the main body of the text, the trends for the 3d ionization features can be inferred from Figure S1 in the Supporting Information.

To sum up, I${}_{3}^{-}$ is negatively charged with a quite diffuse wave function, while iodine atoms are, on their own, very polarizable. Both facts evidence the importance of the inclusion of solvent effects at least at the PCM level to represent the electronic structure and associated ionization spectra of the I${}_{3}^{-}$ solute. The largest effect of the PCM is on SU bands, shifting them closer to the main lines, which is rationalized by the most diffuse nature of the $\sigma^{*}$ orbital. The effect on the main transitions is less pronounced, which in most cases results in a smaller separation of individual main transitions.

### 3.4. Comparison of the Active Spaces and Inclusion of Dynamic Correlation

Application of multi-reference methods to I${}_{3}^{-}$ requires particular care, with the calculations being particularly demanding in terms of the need for human control. In particular, the stability of the composition of the active space is an associated crucial issue. The orbitals at the RASSCF level are optimized, and often the desired orbitals are rotated out of the active space, thus, deteriorating the direct comparison of results for, e.g., different geometries. For instance, in our case, atomic 5s orbitals tend to “intrude” in the active space, superseding the important 5p orbitals. Although the slight sp hybridization is present, one should manually check that a preselected set of molecular orbitals has the desired atomic contributions. This fact complicates the application of RASSCF and RASPT2 methods for points sampled from an MD trajectory in an automated way. Nevertheless, such a procedure has been applied before [8]. In this previous work, only ionization energies were calculated and only 1h determinants were included in the configuration space, thus, improving the convergence and stability of the active space. However, our experience shows that even in such cases, manual control of the quality of the results is mandatory. In the present work, the results were obtained for a smaller number of points in the structure library and systematic geometry scan, where we can notably ensure their reliability.

The valence PES was calculated for the different I${}_{3}^{-}$ geometries with the two active spaces Val_Medium and Val_Full. The comparison of results for both the RASSCF and RASPT2 calculations shows that including all *p* orbitals does not lead to large changes, except for in the total energy shifts (not shown). The only significant difference between the spaces occurs at about a −13 eV BE, outside of the region which can be validated by the experimental results. As the active spaces differ only by the inclusion of doubly-excited configurations, i.e., 3h2p SUs, we concluded that they do not play a role in the region of interest covered by the experiment. Since no large changes were observed, we used the Val_Medium active space as the main electronic structure representation for the valence spectra. In the Supporting Information Figure S2, one can find results for the RASSCF method in the same active space as in Figure 3, which allows the effect of perturbation theory on the valence spectra to be assessed. The global shift applied to all geometries in the RASSCF calculations is −1 eV, while for the RASPT2 approach no common global shift was applied to check against the experiment. No systematic relative shifts between the different geometries are observed for the RASSCF calculations, while the RASPT2 valence spectra tend to be blue-shifted upon symmetry lowering, as discussed in Section 3.2.

**Figure 6:**
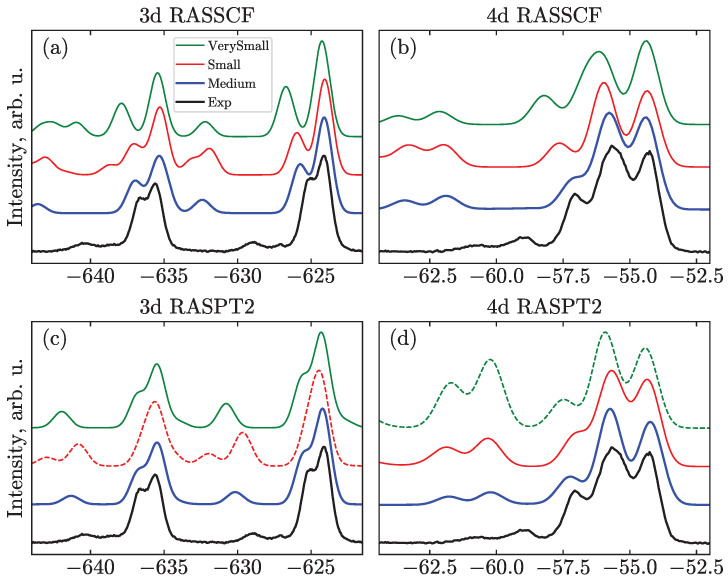
Comparison of active spaces exemplified for 3d (**a**,**c**) and 4d (**b**,**d**) PES using Sym geometry at the RASSCF (**a**,**b**) and RASPT2 (**c**,**d**) levels of theory. Some unsystematic results are shown as dashed lines for illustration: (green dashed line in panel (**d**)) XMS version of RASPT2 with N_VerySmall space demonstrates overestimation of SU intensities; (red dashed line in panel (**c**)) Spurious peaks due to overestimated double SUs for RASPT2 and M_Small space appear at ≈−632 and −643 eV.

An illustration of the effect of the active space choice on the core-ionization spectra for the Sym structure is shown in Figure 6. We do not show the M_Full and N_Full calculations here since they are still computationally demanding, and neither reliable nor systematic results were obtained due to the problems described above. For the same reason, for the Small and VerySmall active spaces, where only three 5p orbitals are included, it is difficult to localize these orbitals and maintain them in the active space. Similarly, systematic calculations for the non-linear geometries could not be performed.

Figure 6 demonstrates that the increase of the active space leads to a decrease of the SU intensities, while leaving the peak positions unaffected. This change brings the simulated spectra into better agreement with the experiments. The effect is even more notable for the main transitions, e.g., increasing the active space from VerySmall to Medium, the separation of the main lines becomes smaller, which agrees better with the experiment.

Regarding the dynamical correlation, i.e., comparing RASSCF with RASPT2, Figure 6, the (c) peaks shift closer to the (i) and (t) ones; the SU transitions, in turn, shift closer to the main peaks. A similar effect was observed for other systems [32] and is also described for the 4d spectra in Ref. [21], where the RASPT2 application leads to a lesser separation between the SU features and 1h transitions, in addition to the overall shift of the spectra to higher BEs. It has to be noted, though, that this is not always the case. For example, see Figure S1, where the Bent RASPT2 structure leads to larger splittings of the main lines and not just to their uniform shift. Additionally, one can notice the shift to lower BEs in the case of the valence spectra (see Figure S2).

In principle, all active spaces are sufficient to qualitatively reproduce all of the I${}_{3}^{-}$ spectral features. However, if we want to semiquantitatively analyze the positions and intensity distributions of the main lines and SU peaks, then one has to use at least the Medium active space and necessarily take dynamic correlation into account. The associated effect can be summarized as follows: adding static correlation (increasing the active space) leads to a better description of the main transitions, whereas including dynamic correlation (RASPT2) in addition brings the SU features into better agreement with the experiment.

It has to be stressed that RASPT2 calculations cannot be considered black box and should not be directly trusted. The so-called intruder state problem manifests itself when the denominator in the energy correction vanishes due to accidental degeneracy of the reference state and the perturbed configuration [29]. One of the solutions in this case is to include such a state in the reference wave function, i.e., to enlarge the active space; this is not always possible or desired, and can potentially cause the appearance of new intruder states. Level shift techniques [27,42] represent another, more robust, remedy, which is systematically applied in this work. In addition, several flavors of RASPT2 have been suggested to cope with this problem. The conventional single-state (SS) version [41], which is used in the current work, gives only second-order energy corrections to the reference states. The multi-state (MS) version [44] modifies wave functions, accounting for the first-order correction and allows for the relaxation of the reference states under the influence of the perturbation. Thus, it is considered to be more precise but inherits problems of SS and adds computational demand. For I${}_{3}^{-}$, the difference between SS- and MS-RASPT2 is negligible for most cases if the calculation can be deemed free from intruder states. Another variant, XMS-RASPT2 [43], substantially overestimates the SU intensities for all the calculated spectra, see the dashed curve in Figure 6d. However, the origin of this overestimation remains unclear. In general, for the studied system, when calculations exhibit irregular behavior— i.e., different types of PT2, different shifts, or different active spaces giving different results—a thorough search for the right solution is needed. One of the ways to judge the reliability of the RASPT2 results is to compare results produced with different active spaces. If the results are notably different, then intruder states may be present (see the red dashed line in Figure 6c, where highly-excited states spoil the spectra).

We conclude that, at least for this very system, it is better not to use PT2 if one cannot guarantee reliability, even if the spectra do not raise any initial suspicions. As an example, one can mention the charge-transfer states found in the 4d PES in Ref. [21]. No evidence of such transitions has been found in experiment [19], despite a careful search. In the calculations presented in this work, where careful analysis of the PT2 results has been performed, such states also never appear, hinting at their spurious nature.

## 4. Discussion

The application of the high-level multi-reference methods to the I${}_{3}^{-}$ system can provide valuable guidance for interpreting the experimental spectra. Nevertheless, we would like to stress one more time that one should be cautious about the quality of the obtained computational results as they can be misleading due to problems with the active space and for the perturbational treatment of dynamic correlation. In this article, we tried to accurately control the reliability of such results, which we believe allows us to produce a more informative interpretation of experiments. However, the need for such control prevented us from performing extensive calculations at many points sampled from the MD simulations. Still, a representative library of structures, including those inferred from previous MD studies [8,9] and experiments [18], as well as the systematic scan through the most relevant region of the ground-state potential energy surface, have been considered in detail.

The primary question, which we tried to answer with this thorough joint experimental/computational study, is about the geometric structure of the I${}_{3}^{-}$ in aqueous solution. The secondary question concerns the differences in the solute structure in the aqueous and ethanol (or other less protic) solvents. Both questions have caused debates in the literature that are not yet closed, with new evidence in favor of controversial (near-linear versus notably bent) interpretations appearing [19,52]. In our previous publication [19] and here, we emphasize the better suitability of the M${}_{4,5}$-edge photoelectron spectroscopy to elucidate both questions than the N${}_{4,5}$-edge, which has been used before. The reason for this is that, in the 3d spectra, both the main and SU bands are more isolated due to larger SOC splitting than in the 4d spectra, where the chemically shifted bands (stemming from non-equivalent atoms) and those split due to SOC largely overlap. However, joint analysis of the valence, 4d, and 3d spectra provides additional arguments about the geometric structural distribution, as the data are complementary to each other.

In view of the main question, from the computational analysis of the ground-state potential energy surface with and without solvent, one concludes that a polarizable medium favors structures with asymmetric bond lengths, with no evidence of flattening of the surface along the bending angle coordinate. Thus, one surmises that a broad distribution of structures should be present in solutions with dominating linear or near-linear species. This finding is supported by the shape of the PES from all considered edges. For instance, from Figure 3 and Figure S3, one infers that the equilibrium geometry should be near-linear with a bending angle of 165–180° and larger bond length of around 3.1–3.15 Å, rather than 3.4 Å, as reported for the Bent geometry, i.e., there is only a slight degree of asymmetry. However, one should not exclude the probability of contributions from strongly bent (153°) structures with slight bond length asymmetry (around 3.15 Å), since the dependence of spectra on the bending angle is only moderate, cf. solid and dotted lines in Figure 4. Next, this conclusion is in accord with the intensity ratios between SUs and main transitions, see Figure 4, where the bond-length difference of 0.15–0.2 Å and near-linear geometries provide the best agreement with experiments.

We note that our results and interpretation disagree with the findings of Kim et al. [18], where the Bent structure (ΔR = 0.45 Å) is claimed to dominate in aqueous solution and, with a recent report [52] from the same group, where approximately the same central angle of 152° is found for methanol solution (ΔR = 0.13 Å, bond lengths are in agreement with our estimations in this case), in contrast to Ref. [18] and without explanation of this disparity. From our calculations (Figure 4), one can see that the 3d photoelectron spectrum would be very different from the experiment for ΔR of more than 0.25 Å. One can argue that the structures obtained from the pump-probe X-ray scattering experiments [18,52] are due to rovibrationally excited fragment species held within a solvent cage or recombined photoproducts. In any case, the error bars of both experiments also seem to span a near-linear geometry. Moreover, in the most recent time-resolved X-ray solution scattering experiments [53], the MD ground-state structures in different solvents of Ref. [9] were used for the analysis of data. We remind the reader that these and similar structures also have modest asymmetries and bending angles, ≥170°, and are in agreement with our findings.

Finally, we note that peak 10 and its origin remain enigmatic, as it is absent in the calculated spectra. It can stem, for example, from an additional hydrogen-bond or other interactions, which increase the SU splitting and shifts the SU feature from the central iodine atom closer to the main lines. We found a trend when such a shift indeed occurred for the increased bond-length asymmetry, see Figure 4. The splitting between the SU contributions is at its maximum for the MD geometry and equals about 1.1 eV (Figure S3), which might be ascribed to the experimental splitting of 1.7 eV between peaks 10 and 11. A possibility for peak 10 to originate from highly-asymmetric geometries, coexisting with more symmetric ones, still remains. However, the 3d spectrum is reproduced already well enough with near-linear geometries; generally more asymmetric ones should have introduced more differences in features of the total spectrum, especially for the main transitions.

## 5. Conclusions

In this work, we have addressed different computational aspects of calculating $I_{3}^{-}$ photoelectron spectra, aiming at a reliable joint experimental/computational interpretation of the anionic geometric structure in aqueous solution. The complex electronic structure of the final states, especially those corresponding to the shake-up ionization transitions, makes it mandatory to account for static and dynamic correlation. However, in performing such calculations, one may encounter numerous problems with variational stability and the convergence of perturbation series. A careful account of this issue allows spurious results to be avoided and increases the efficiency of the theory in assisting the interpretation of experimental data.

Armed with this knowledge, we analyzed the $I_{3}^{-}$ geometric structure with a particular focus on the M_4,5_-edge (3d) PES, being the most informative among the here-studied edge spectra. From the analysis of the ground-state potential energy surface, positions, and intensity ratios of main and especially shake-up ionization features, we conclude that the spectra of near-linear anion structures, with moderate bond length asymmetry, agree best with the experimental results. This, however, does not close the debate on the intriguing question of the structure of this model system, as other techniques suggest notably larger bending angles and bond asymmetries [18,52], suggesting further studies are required.

We hope that our work stimulates further ab initio studies and guides spectral calculations on aqueous triiodide and similar systems. These studies pave the way for studying the liquid-phase dissociation dynamics of $I_{3}^{-}$ with PES upon electronic excitation, since the associated spectra will also be sensitive to changes in anionic bond lengths and angles.

## Figures and Tables

**Table 1:** I${}_{3}^{-}$ geometric structures considered in the present study and their symmetries; the actual point symmetry group used in the presented calculations is the highest common symmetry, $C_{s}$. Different geometries are ordered according to their decreasing symmetry from top to bottom.

Label	Type	Symmetry	*r*(I–I), Å	ΔR, Å	*∠*(I–I–I), °	References ${}^{1}$
Sym	Symmetric linear	$D_{\infty h}$	2.91, 2.91	0.00	180	CASPT2 vacuum [23]
Lin	Linear, slight asymmetry	$C_{\infty v}$	2.94, 3.09	0.15	180	Exp in methanol [18]
MD	Near-Linear	$C_{s}$	2.90, 3.10	0.20	171	MD simulations [9]
HBent	Bent, slight asymmetry	$C_{s}$	2.94, 3.10	0.16	165	Intermediate point
Asym	Linear, max asymmetry	$C_{\infty v}$	2.82, 3.37	0.55	180	MD simulations [8]
Bent	Asymmetric bent	$C_{s}$	2.93, 3.38	0.45	153	Exp in water [18]

^1^ In some cases, geometries do not exactly coincide with the references provided but are close to them.

**Table 2:** The number of the final states of different multiplicities considered for different active spaces. The number of valence states supported by these active spaces is given in parentheses. Imaginary shifts used for RASPT2 calculations (see the main body of the text) are also given.

Active Space	Doublet	Quartet	Imaginary Shift, a.u.
Val_Full	240	–	0.1
Val_Medium	72	–	0.1
N_Full, M_Full	1455 (240)	624 (84)	0.2
N_Medium, M_Medium	327 (72)	148 (28)	0.2
N_Small, M_Small	133 (8)	46 (1)	0.2
N_VerySmall, M_VerySmall	81 (6)	31 (1)	0.2

## Data Availability

The data presented in this study are available on request from the corresponding author.

## Supplementary Materials

The following supporting information can be downloaded at: https://www.mdpi.com/article/10.3390/molecules28145319/s1, Figure S1: Comparison of RASSCF and RASPT2 M_5_ spectra for different geometries and solvents; Figure S2: Systematic presentation of M_4,5_, N_4,5_, and valence PES for different geometries at the RASSCF level; Figure S3: Assignment of MD and Bent spectra; Figure S4: SU-to-main peak intensity ratios for the 165° bending angle; Figure S5: influence of the PCM parameters on the spectra.

## Abbreviations

The following abbreviations are used in this manuscript:

BE	Binding Energy
CASPT2	Complete Active Space Second-Order Perturbation Theory
FCI	Full CI
MD	Molecular Dynamics
MO	Molecular Orbital
MS-RASPT2	Multi-State RASPT2
PES	Photoelectron Spectrum
PCM	Polarized Continuum Model
RASPT2	Restricted Active Space Second-Order Perturbation Theory
RASSCF	Restricted Active Space Self–Consistent Field
SOC	Spin–Orbit Coupling
SU	Shake-Up
SS-RASPT2	Single-State RASPT2
XMS-RASPT2	X Multi-State RASPT2
