# Supplementary material for: Valence and Core Photoelectron Spectra of Aqueous I3− from Multi-Reference Quantum Chemistry
Source: Molecules. 2023 Jul 10;28(14):5319. doi: 10.3390/molecules28145319 (PMC10383688; doi:10.3390/molecules28145319)
Supplement: Supplementary file 1 [file molecules-28-05319-s001.zip › molecules-2464872-supplementary.pdf]

Supplementary material
Valence and core photoelectron spectra of aqueous I_3^- from multi-reference quantum chemistry

Vladislav Kochetov[1], Md Sabbir Ahsan[2,3], Dennis Hein[4,5], Iain Wilkinson[2], and Sergey I. Bokarev[6]

[1]Institut für Physik, Universität Rostock, Albert-Einstein-Str. 23-24, 18059 Rostock, Germany
[2]Helmholtz-Zentrum Berlin für Materialien und Energie, Hahn-Meitner-Platz 1, D-14109 Berlin, Germany
[3]Department of Physics, Freie Universität Berlin, Arnimallee 14, D-14195 Berlin, Germany
[4]Operando Interfacial Photochemistry, Helmholtz-Zentrum Berlin für Materialien und Energie, Hahn-Meitner-platz. 1, D-14109 Berlin, Germany
[5]Department of Physics, Humboldt-Universität zu Berlin, Newtonstrasse 15, D-12489 Berlin, Germany
[6]Chemistry Department, School of Natural Sciences, Technische Universität München, Lichtenbergstr. 4, 85748 Garching, Germany

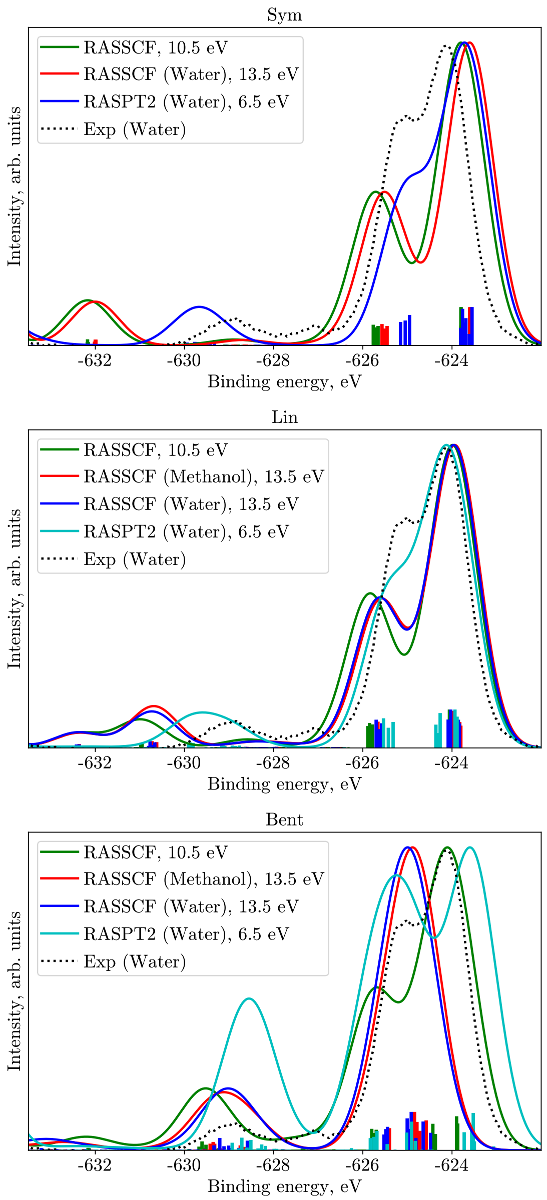

Figure S1. Comparison of the RASSCF and SS-RASPT2 M_5 spectra for the **Sym** (top), **Lin** (middle), and **Bent** (bottom) structures in vacuum, methanol, and water. The **M_Medium** active space was used. For the **Sym** and **Lin** species, the difference in results is rather small, apart from the overall spectral shifts, when comparing the water, methanol, and vacuum spectra. For the **Bent** geometry, the difference between different solvents is more notable, especially in the stick spectra, but is masked in the convoluted ones.

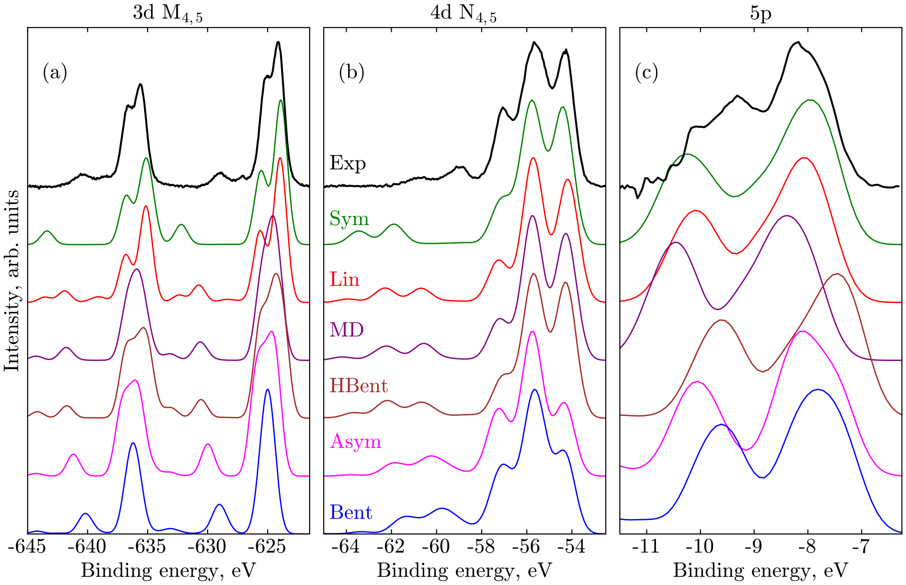

Figure S2. PESs at the RASSCF level for different geometries: (**a**) 3d with the **M_Medium** active space (global shift 13.5 eV, Gaussian broadening FWHM 1.25 eV) (**b**) 4d with the **N_Medium** active space (shift 2.8 eV, broadening 1.05 eV), (**c**) 5p valence with the **Val_Medium** active space (shift -1 eV, broadening 0.85 eV)

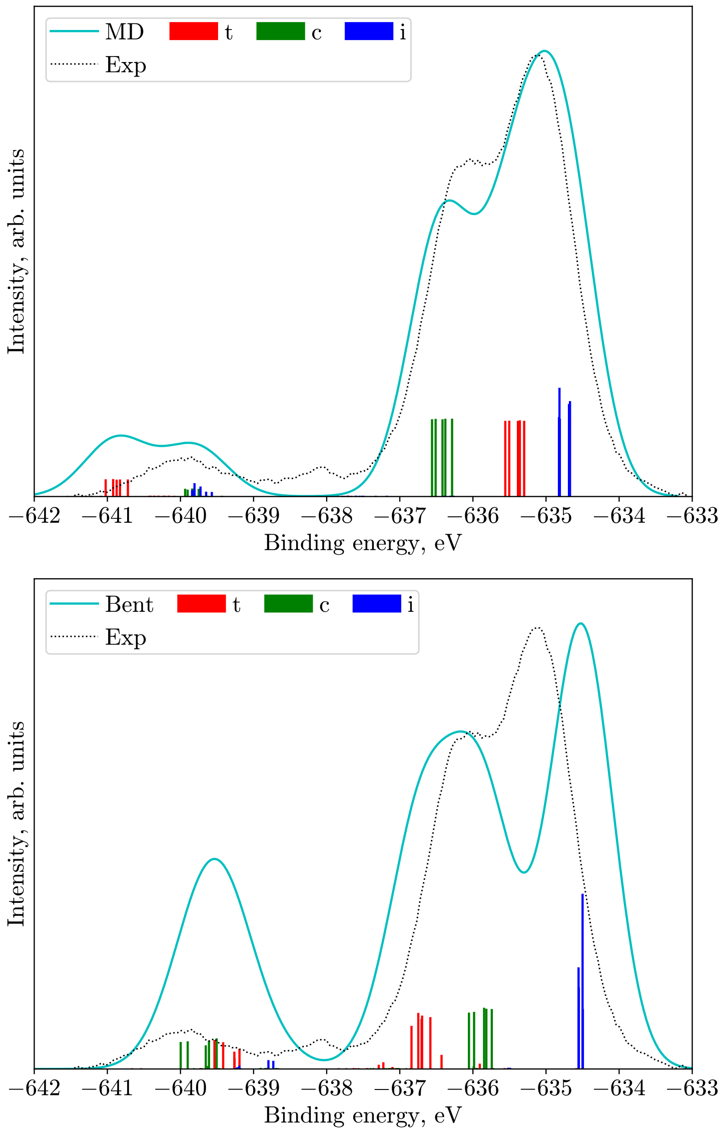

Figure S3. SS-RASPT2 M_5 spectra for the **MD** and **Bent** geometries as candidates to fit to the experimental results. The former is in better agreement with the experiments for the main transitions and for the SU splitting. The stick spectra are colored according to the hole-bearing atom, as deduced from a Mulliken charge analysis. In the broadened spectra, the FHWM is reduced to 1 eV for better visibility of the split SU feature.

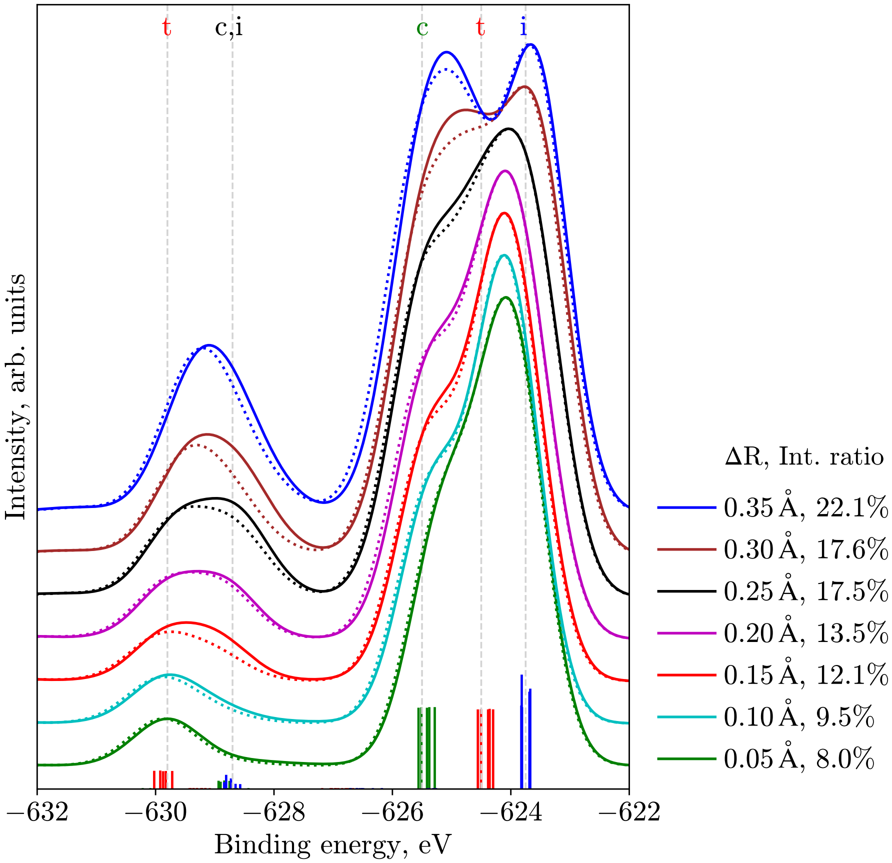

Figure S4. SS-RASPT2 M_5 spectra calculated for a fixed bond angle of 165° and varied bond length. Dotted lines are given for comparison with the spectra calculated for an I–I–I angle of 180°, which are shown in the main text with solid lines. The legend shows the difference i n b ond l engths a nd SU-to-main peak intensity ratio for the 165° bending angle. The experimental value for this ratio for the 3*d* spectrum in an aqueous solution is 12.2%.

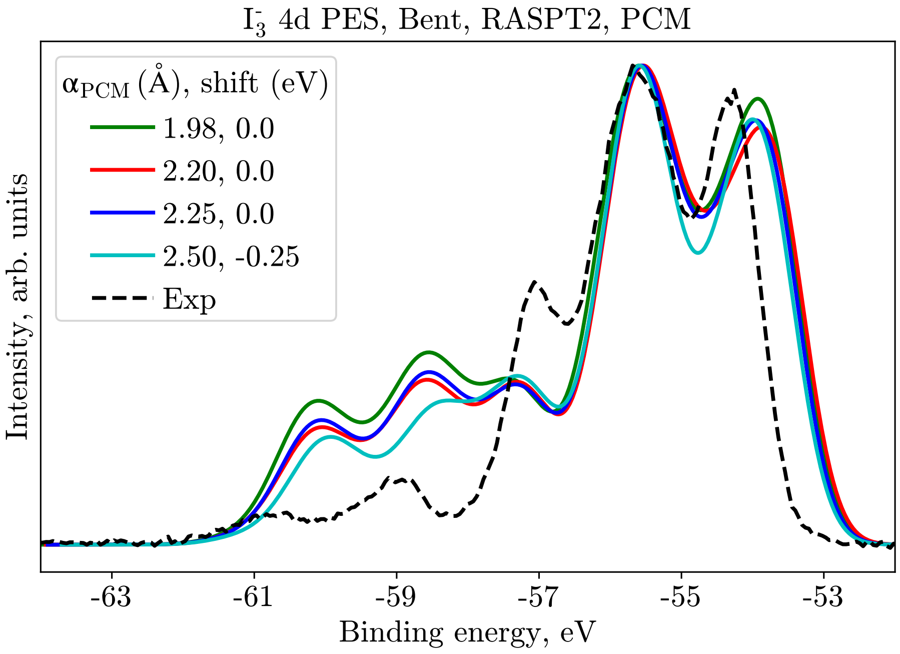

Figure S5. SS-RASPT2 $N_{4,5}$ spectra calculated for different radii α_{PCM}, used to construct PCM cavities for the **Bent** geometry.
